# From burden to action: Saudi Arabia’s strategy on antimicrobial resistance

**DOI:** 10.1515/med-2026-1380

**Published:** 2026-04-27

**Authors:** Shuaibu Abdullahi Hudu, Abdulgafar Olayiwola Jimoh

**Affiliations:** Center for Health Research, Northern Border University, Arar, Saudi Arabia; Department of Microbiology, Faculty of Medicine, Northern Border University, Arar, Saudi Arabia; Department of Pharmacology and Therapeutics, Faculty of Basic Clinical Sciences, College of Health Sciences, Usmanu Danfodiyo University, Sokoto, Sokoto State, Nigeria

**Keywords:** antimicrobial resistance, One Health, Saudi Arabia, stewardship, surveillance

## Abstract

Antimicrobial Resistance (AMR) is a significant public health challenge in Saudi Arabia and globally. This review consolidates evidence on the burden, drivers, policy responses, and progress in controlling AMR from 2010 to 2025 across human health, animal health, and the environment. The burden is substantial, especially from multidrug-resistant strains such as ESBL-producing Enterobacteriaceae, carbapenem-resistant *Klebsiella pneumoniae*, and methicillin-resistant *Staphylococcus aureus*. Factors contributing to this include antibiotic misuse, over-the-counter availability, mass gatherings, insufficient infection control, and agricultural use. Saudi Arabia has made progress through a One Health-oriented National Action Plan (2017, renewed 2022–2025), antimicrobial stewardship programs, surveillance efforts, and stricter regulations. However, challenges remain in community surveillance, laboratory capacity, and behavioral change. As indicated by key stakeholders, further efforts to expand stewardship, improve diagnostics, regulate antibiotic sales, and implement AI-driven surveillance are essential next steps. Increased investment in research, innovation, and partnerships can position Saudi Arabia as a leader in regional AMR containment initiatives.

## Introduction

Antimicrobial resistance (AMR) is one of the most serious public health challenges, posing a threat to modern medicine and returning us to the pre-antibiotic era after decades of progress in treating infectious diseases. AMR has been classified as one of the top 10 health threats to humanity and demands a collaborative, immediate response [1]. If left unchecked, AMR is expected to be responsible for a death toll of up to 10 million people per year by 2050, which would exceed the mortality from cancer and other significant diseases combined [2]. This alarming trend highlights the urgent requirement for international, evidence-based interventions and geographical containment of resistant organisms.

The global burden is uneven, with some regions at greater risk. The Middle East is a hotspot due to the high demand for antibiotics, which are used without appropriate monitoring, thereby accelerating the selection and spread of resistant organisms [3]. Further complicated by health-care system hurdles, such as overcrowding and staffing shortages. These factors are fostering the spread of resistant infections. In this regional landscape, Saudi Arabia occupies a central role. As a major destination for religious pilgrimage, the country welcomes millions of pilgrims each year to perform Hajj and Umrah. Although a mass religious gathering, these events also represent a significant threat to the cross-border import and export of multidrug-resistant organisms and complicate efforts for AMR containment, which can be defined as “The development, control, adequacy of supply and proper use of antimicrobials” [4], 5].

There is a significant burden of AMR in Saudi Arabia. Representative national data have disclosed alarmingly high incidence rates of key resistant pathogens, including extended-spectrum β-lactamase (ESBL)-producing Enterobacteriaceae, carbapenem-resistant *Klebsiella pneumoniae*, and methicillin-resistant *Staphylococcus aureus* (MRSA), which are frequently above the international burden [6], 7]. The factors driving this resistance are complex and rooted in the country’s clinical, social, and environmental landscape. Main factors have been the misuse and/or overuse of antibiotics in hospitals and communities, the continuation of over-the-counter sales despite regulations, substandard infection prevention and control (IPC) practices in healthcare facilities, and the widespread use of antimicrobials in agriculture and veterinary medicine [8], 9].

Realizing the severity of the threat, the Saudi government has shown a strong commitment to an organized national approach. This effort resulted in the first National Action Plan on AMR, issued in 2017 and re-enacted through 2022–2025. These are in accordance with the WHO Global Action Plan and recommend a multi-sectoral “One Health” approach that involves human, animal, and environmental health sectors [10]. Cornerstone measures include implementing antimicrobial stewardship programmes (ASPs), improving national surveillance structures, and reinforcing regulatory frameworks. However, considerable challenges remain, including the lack of community-level surveillance, regional inequalities in laboratory capacity, and the need for continued perceptual and behavioural change among healthcare workers and private individuals [11], 12].

Hence, the current review aims to consolidate existing evidence on the burden, drivers, and intervention strategies of antimicrobial resistance in the Kingdom of Saudi Arabia. The concerted use of antimicrobial stewardship, surveillance, and regulation across One Health sectors has led to observable success in the containment of AMR. The main research question this review addresses is: How effective are Saudi Arabia’s integrated policy, stewardship, and surveillance strategies in controlling antimicrobial resistance across human, animal, and environmental sectors? It also outlines a forward-looking roadmap for improving AMR control, aligned with the Saudi national action plan (2022–2025) and global health security goals.

## Methodology

A narrative review on AMR in Saudi Arabia was conducted, which discussed burden, drivers, and risk factors of AMR, stewardship and policy, and strategic interventions. A comprehensive search of the databases, including PubMed, Scopus, Web of Science, and Google Scholar (2010–2025), using MeSH terms and keywords related to AMR, stewardship, policy-making, in addition to Saudi Arabia for Hajj and Umrah, also included One Health. The eligible types of studies were original research, review articles, and national-level reports on AMR at the human, animal, or environmental levels, written in English or Arabic. Grey literature, such as Ministry of Health reports and National Action Plans (2017, 2022–2025), WHO publications, and conference proceedings, was also reviewed. Epidemiological, resistance patterns, stewardship programs, risk factors, and policy framework data were extracted and narratively synthesized. Quality assessment comprised study design, representativeness, and reporting transparency. Limitations include potential publication bias, heterogeneity of surveillance methods, and limited capture of local or regional interventions. This study was based exclusively on publicly available data and published literature. Therefore, ethical approval and informed consent were not required.

### Burden of AMR in Saudi Arabia

**Figure 1:**
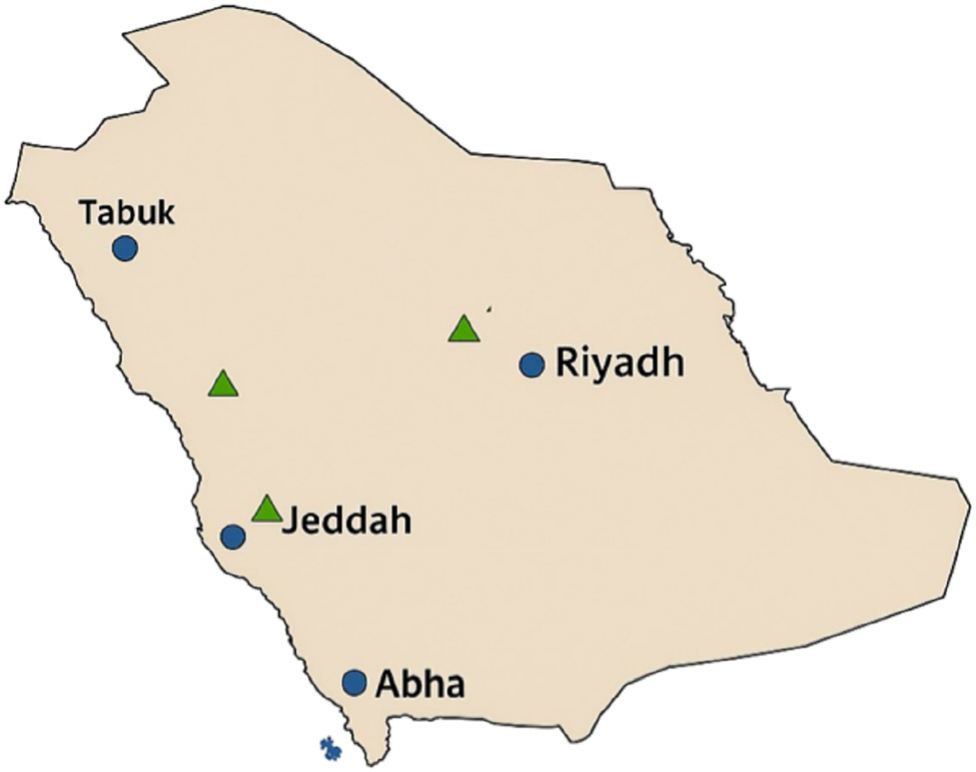
National monitoring centers for antimicrobial resistance in Saudi Arabia (by 2025). This map shows the locations of national AMR surveillance sentinel sites managed by the ministry of health and sponsored by the National AMR committee. Blue circles=hospital surveillance sites. Green triangles=environmental or veterinary surveillance sites of the ministry of environment, water, and agriculture.

In the present study, we found that Saudi Arabia’s coordinated One Health-driven antimicrobial stewardship policies, strengthened surveillance systems, and regulatory interventions have contributed to reductions in inappropriate antimicrobial use and improvements in resistance containment. However, essential gaps persist in community surveillance and environmental monitoring. The threat of AMR in Saudi Arabia is no longer seen only as an emerging public-health problem. A recent meta-analysis of 25 studies identified disturbingly high pooled prevalence estimates for several important resistance phenotypes, including approximately 38.7 % for CRE, approximately 26.4 % for extended-spectrum beta-lactamase (ESBL)-producing Enterobacteriaceae, and approximately 15.2 % for MRSA across clinical, environmental, and foodborne habitats [6]. These values are much higher than most international averages and suggest pan-institutional carriage of resistant organisms. This burden is also reflected in surveillance data from single centers. For example, at KFMC in Riyadh, ESBL rates for *Escherichia coli* and *K. pneumoniae* were rising steadily across some years: the overall combined figures for ESBL positivity thereof were some 20.8 % among all these combined isolates; again, *E coli* showed sustained increases from about 32 %, already up towards something like 40–41 % over multiple years [13]. In the western region (Mecca and Jeddah), ESBL occurrence among GNB is approximately 30 % in some studies; however, considerably lower ESBL production rates of 6–25 % are reported from other regions, indicating geographical variability [14]. Regional differences in AMR prevalence are evident across surveillance sites in Saudi Arabia (Figure 1).

For *K. pneumoniae*, the situation is especially worrying. A recent hospital‐based study including 944 *K. pneumoniae* isolates reported that as many as 57 % were multidrug resistant (MDR), of which 37 % were ESBL producers. In contrast, approximately 20 % were carbapenem‐resistant [15]. Because *K. pneumoniae* is a significant cause of both nosocomial and community‐onset infections, such as UTIs, pneumonia, and bloodstream infections, these levels of resistance greatly restrict the available agents for empiric therapy and are associated with worse outcomes.

UTIs also demonstrate high resistance rates. In Southwestern Saudi Arabia, *E. coli* and *K. pneumoniae* are the most common gram-negative organisms. Still, in a cross-sectional study, 36 % of uropathogen isolates were multidrug-resistant, possessing one or more known AMR phenotypes [16]. This mirror concerns hospitals, highlighting that a burden exists outside tertiary care and ICU facilities as well. From a consumption perspective, data reflect a mixture of high and unbalanced antibiotic use, with a preference for broad-spectrum classes. In research on outpatient prescriptions from Saudi Arabia, approximately 40.8 % of the total number involved “Watch” group antibiotics by the WHO AWaRe classification system, whereas 59.2 % fell into the “Access” group [17]. Of those prescriptions, antibiotics including amoxicillin–clavulanic acid and ciprofloxacin were most frequently used. Lippincott Journals. Further, a point‐prevalence survey of 26 ministry‐of‐Health hospitals in 2016 reported that 46.9 % of all inpatients received at least one antibiotic; third-generation cephalosporins were the most commonly prescribed class (17.2 %), and respiratory tract infections were the most common indication [18]. The pathogen-specific prevalence of resistance across primary clinical isolates is summarized in Table 1.

**Table 1:** Pathogen-specific resistance prevalence in Saudi Arabia (pooled national data, 2020–2024). Data are pooled synthetic national estimates from regional surveillance and reports (2019–2024). MRSA and ESBL prevalence are highest in large metropolitan hospitals, and carbapenem resistance in *Klebsiella pneumoniae* and *Acinetobacter baumannii* is among the most difficult-to-treat organisms.

Pathogen	Key resistance phenotype	Percentage resistant (median, IQR)	Source setting	Comments	Reference
*Escherichia coli*	ESBL-producing	34.8 % (28.5–40.1)	Hospital and community	Increasing trend since 2018; high in urinary isolates.	[19]
*Klebsiella pneumoniae*	Carbapenem-resistant	19.6 % (15.0–24.3)	Tertiary care	Concentrated in ICU and surgical wards.	[20]
*Acinetobacter baumannii*	MDR (≥3 drug classes)	48.3 % (42.0–55.0)	Hospital	Linked to ventilator-associated infections.	[21]
*Pseudomonas aeruginosa*	Carbapenem-resistant	22.7 % (18.9–27.1)	Hospital	Declining trend post-ASP rollout.	[22]
*Staphylococcus aureus*	MRSA	14.9 % (12.5–17.3)	Hospital and community	Declining since 2020 due to infection control.	[23]
*Enterococcus faecium*	Vancomycin-resistant	8.3 % (5.7–10.4)	Hospital	Detected mainly in ICU bloodstream isolates.	[24]
*Salmonella spp.*	Ciprofloxacin-resistant	7.1 % (5.2–8.6)	Foodborne	Low but emerging in poultry-associated strains.	[25]

ESBL, extended-spectrum β-lactamase; MDR, multidrug-resistant; MRSA, methicillin-resistant *Staphylococcus aureus*; ICU, intensive care unit; IQR, interquartile range.

Policy responses have also been effective in certain areas. For example, since the national restriction policy (2017–2019), total antimicrobial sales decreased by 23.2 %, from 818.9 million SAR to 648.4 million SAR. [26]. It is worth noting that, based on the Access group, sales of such drugs as amoxicillin even dropped by 70 % over this period. Still, there were drugs, including combination antibiotics like amoxicillin and clavulanic acid, that rose [26]. Collectively, these data demonstrate that KSA is experiencing a high and rising burden of AMR across a range of pathogens and care settings, with high antibiotic use across the spectrum, often including broad‐spectrum agents. Critical gaps, however, persist with inconsistent data availability across regions, relatively scarce community‐based surveillance, and published documentation of burdens (clinical burden, economic costs) beyond single-centre-based studies.

### Risk factors and strategic solutions for antimicrobial resistance in Saudi Arabia

Preventing antimicrobial resistance must be based on harmonized interventions across human health, animal health, the environment, and the healthcare system under the One Health approach. Fundamental interventions include enhancing antimicrobial stewardship programs, prescribing antibiotics only on prescription, extending immunization coverage, and raising public awareness to avoid unnecessary antibiotic use in human health. In the livestock sector, measures to reduce non-therapeutic use of antimicrobial drugs, encourage vaccination and biosecurity, and ensure that veterinary prescribing schemes are in place should be implemented. Environmental interventions include wastewater treatment, surveillance of resistant organisms in aquatic environments, and minimization of pharmaceutical pollution. In healthcare settings, good infection control practices should be implemented, and surveillance should be aligned with healthcare-associated infections. A combination of stewardship and diagnostics is crucial to prevent transmission and selection of resistant pathogens [27], 28].

Hospitals, as a significant reservoir for the development and dissemination of antimicrobial-resistant pathogens, require robust IPC practices. Basic components of IPC measures include strict hand hygiene, environmental services such as cleaning and disinfection, patient isolation with cohorting as applicable, and implementation of device-associated prevention bundles. The measurement of multidrug-resistant organisms and their integration into antimicrobial stewardship programs and staff education have decreased hospital-acquired spread [29], 30]. The concurrent use of IPC and stewardship has been shown to reduce healthcare-associated infections, antimicrobial consumption, and the transmission of resistant organisms within hospitals.

These patients often need prolonged or broad-spectrum antibiotic regimens, which favour the emergence of multidrug-resistant organisms. Pathogens, including extended-spectrum beta-lactamase (ESBL)- producing *E. coli*, carbapenem-resistant *Acinetobacter baumannii*, and methicillin-resistant *S. aureus* (MRSA), are increasingly being described in Saudi hospitals [31]. Advances in Technology have made us more susceptible to antimicrobial resistance. Dietary Habits Re-routing Susceptibility Burden of Communicable as well as NCDs will be tremendous. The potential for delays in laboratory confirmation or a lack of access to rapid diagnostics would lead the clinician to prescribe antibiotics as parenteral treatment, thereby perpetuating the resistance problem. Improving Antimicrobial Stewardship Programs (ASPs) is thus paramount. They suggest that hospitals need to have evidence-based prescribing protocols, incorporate clinical decision support tools, and conduct regular audit-and-feedback programs. Technological innovations such as point-of-care molecular testing and susceptibility assays enable targeted therapy while minimizing unnecessary exposure to broad-spectrum antibiotics. Regular re-education of health care providers on current national guidelines and emerging resistance patterns is also crucial.

Community antibiotic use is mediated mainly by behavioural and sociocultural factors [8]. Non-prescription use of remaining antibiotics, patient demand for unnecessary antibiotics for minor infections, and noncompliance with prescriptions are standard practices that drive selective pressure for resistant strains and escalate AMR outside the healthcare sector [32]. Mass gatherings, such as the regular Hajj and Umrah pilgrimages, offer a specific behavioral risk. The millions of pilgrims gather in a small geographic area, leading to an increase in respiratory and gastrointestinal infections and frequent antibiotic use. The spread and International dissemination of MDR pathogens among pilgrims have been documented [5]. These behavioral motivations would need to be targeted in culturally sensitive public education campaigns designed to counter self-medication on a large scale, focusing particularly on the risks of self-treatment and incomplete courses prescribed by a healthcare provider. Preventative measures such as pre-travel information, vaccination programmes, hygiene promotion, and post-event monitoring can reduce the spread of infections and inappropriate antibiotic use at large gatherings [33], 34]. Behavioural science should be incorporated into public health messaging to improve adherence and the long-term impact on spread.

Environmental and agricultural activities are also major contributors to AMR [9], 35]. Antibiotic use in farm animals for therapeutic, growth promotion, and prophylactic purposes in poultry, cattle, and aquaculture is widespread [36], 37]. Uneven application of veterinary laws and contamination of the environment with antibiotic-resistant gram-positive bacteria via wastewater or surface artesian are promoting the transfer of resistance genes to human pathogens. Bacterial communities present in soil, water, and foodstuffs become reservoirs of resistant strains that sustain two forms of nature-acquired resistance: hospital-acquired resistance and community-acquired resistance [38]. It is thus crucial to operationalize the OH concept. By focusing on the monitoring of the use of antimicrobials in agriculture, strict compliance with established residue standards for food products and environmental measures can minimize contamination. Encouraging a reduction in the use of antimicrobials by promoting alternative strategies for disease prevention in livestock, such as vaccination and improvements in biosecurity and husbandry practices, can reduce reliance on these drugs while maintaining productivity.

There have always been such systemic and regulatory shortcomings in anticipation of inappropriate antibiotic use. Access to antibiotics over the counter, lack of uniform stewardship practices, and adherence to infection prevention and control (IPC) in health facilities remain substantial systemic risks [39], 40]. Restricted laboratory capacity and a disjointed surveillance system prevented early identification of perpetrators, allowing resistant strains to spread unchallenged. The reinforcement of laws and regulations, such as the prohibition of non-prescription sales, the regular monitoring of pharmacy and veterinary practices, and sanctions, is the imposition of necessary. Broadening laboratory infrastructure and linking standardized surveillance systems allow timely identification of resistant pathogens, and incorporating stewardship and IPC actions into national accreditation standards will promote the harmonized implementation across health facilities.

International travel additionally increases the threat of AMR in Saudi Arabia. The fact that the country is a hub for international travel, medical tourism, and expat workers contributes to this burden of resistant organisms. Patients may bring back resistant organisms and introduce them into local healthcare settings when traveling, whereas travel by healthcare workers (HCWs) can inadvertently spread MDR organisms. External introduction, by residents and visitors alike, of resistant strains can be minimised through pretravel screening, vaccination, and health education. Enhancing global coordination, information exchange, and regional networks for surveillance will enable early detection and a coordinated response to new threats.

The interlinkages of these risk factors call for an integrated, holistic response. Critical approaches include standardizing ASPs across healthcare facilities and incorporating rapid diagnostics in addition to audit-feedback mechanisms; developing culturally tailored public education campaigns to promote rational belief and behavior *vis-à-vis* antibiotics; moving toward operationalizing One Health for interventions that span human, animal, and environmental health; reinforcing regulatory and policy actions, including prescription enforcement as well as accreditation-linked stewardship efforts; investment in surveillance alongside laboratory capacity sufficient to track evolving resistance patterns in real time; deployment of digital-innovations like artificial intelligence (AI) for outbreak detection purposes along with clinical decision support tools overall informatics for disease surveillance [41] with clear direction on how these innovations will be achieved at the national level and addressing mobility-related risks at both mass-gathering events through specific screening interventions such as vaccination and syndromic surveillance alongside medical tourism using a cross-border approach supported by focused surveillance.

Overall, the determinants of AMR in Saudi Arabia are multi-factorial and interconnected, including clinical practices, social habits, environmental exposure, health system shortfalls, and international movement. A coordinated, multisectoral response in accordance with the One Health approach and the Saudi National Action Plan is needed. Through applying focused interventions, strengthening control, and harnessing technological and behavioral advances, Saudi Arabia can control the emergence and transmission of resistant pathogens, protect public health, and support specific regional and global AMR containment activities.

### Antimicrobial resistance policy context and control

**Figure 2:**
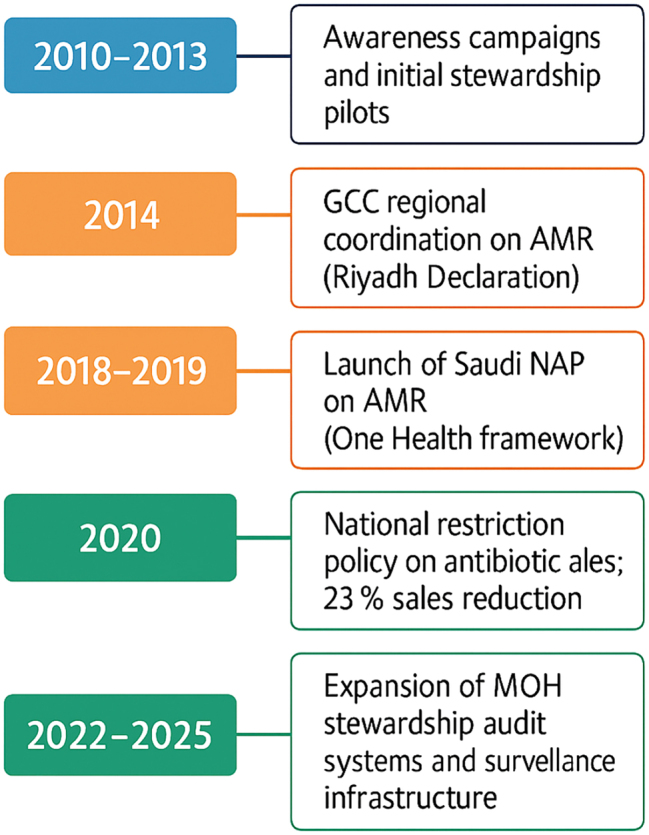
Timeline of antimicrobial resistance (AMR) policy milestones in Saudi Arabia, 2010–2025. The figure presents a vertical timeline of Saudi Arabia’s progress in national AMR policy development from 2010 to 2025.

The control of AMR in Saudi Arabia is guided by a staged, tiered system that incorporates national policy frameworks, inter-ministerial cooperation, and regulatory surveillance to combat this critical public health problem. In the early 2010s, initial attempts mainly aimed to raise awareness and start hospital-based stewardship programs. National campaigns directed at health care professionals and the public about the dangers of antibiotic misuse and abuse, as well as the rising threat of antibiotic resistance in our global society. At the same time, some hospitals followed suit with periodic antimicrobial stewardship programs (ASPs) to enhance prescribing practices and decrease the incidence of resistant organisms. Although these early initiatives paved the way for organised stewardship, they remained largely fragmented and heterogeneous, with no uniform scope or degree of impact across regions [11]. The evolution of Saudi Arabia’s AMR response has progressed through several strategic phases (Figure 2).

A more systematic national response took shape in 2017 with the launch of Saudi Arabia’s inaugural holistic national action plan (NAP) on AMR. The strategy was consistent with the World Health Organization’s Global Action Plan on AMR and focused particularly on the adoption of One Health principles that acknowledge human, animal, and environmental health-based influences on the development of resistance. The first NAP addressed the establishment of surveillance systems, the promotion of the rational use of antimicrobials in human and veterinary medicine, the expansion of laboratory capacity, and research to underpin further decision-making [10]. It established specific goals for hospitals, primary care, and public health institutions, contributing to the institutionalization of stewardship initiatives and the systematic surveillance of defined daily doses [38], 42].

Subsequent versions of the NAP, from 2022 to 2025, have woven Saudi Arabia’s dedication to a coordinated cross-sectoral response into the document. The revised plan emphasized the need for rapid expansion of national surveillance networks, the integration of antimicrobial stewardship in all levels of healthcare facilities, and the development of regulatory frameworks to restrict over-the-counter sales. The revised NAP, notably, highlighted One Health concepts, recognizing that the use of AMU in agriculture, food production, and veterinary medicine is a major driver for selection and spread of resistant pathogens. The introduction of these components marked an evolution in Saudi Arabia’s response to AMR, from awareness-raising activities and sporadic interventions to the development of a comprehensive, evidence-based national strategy [7].

Control system for AMR in Saudi Arabia. The control structure is marked by a centralized MOH that plays the leading role in policy-making, supervision, and coordination across the health sector. The latter is concerned with drafting national guidelines, overseeing drug use, and assessing the effects of stewardship programmes in acute care and primary healthcare settings. To ensure effective cross-sectoral collaboration, an inter-ministerial committee was formed, comprising the Ministry of Environment, Water and Agriculture, the Saudi Food and Drug Authority, and regulatory authorities for veterinary products. This committee enables the integration of surveillance, the alignment of regulations, and the coordination of regulation and messaging on antimicrobial use across human health, veterinary medicine, and environmental management. The intuition behind the control structure is that system-oriented AMR control demands coordinated action across different sectors.

The National AMR Committee is therefore a key operational instrument in this context. The committee plans surveillance, standardizes information collection, and follows national guidelines in public and private healthcare facilities. It also measures trends in antimicrobial resistance and consumption, producing evidence to help tailor policies and interventions. In addition, the committee’s responsibilities include regulatory authority, such as monitoring the use of prescription-only antibiotics, building laboratory capacity, and generating public awareness campaigns. Centralization of these roles improves accountability and facilitates prompt decision making and thus maximizes the overall operational efficiency of the national AMR plan [31], 43].

The Saudi Arabian strategy also incorporates regulatory and legislative components to reduce the misuse of antimicrobials. Such regulations banning over-the-counter sales of antibiotics have been introduced in human and animal medicine, and enforcement has been implemented, but not without issues and national compliance problems. The Saudi Food and Drug Authority’s licensing and inspection frameworks are essential for monitoring compliance, with penalties acting as a disincentive. In addition, hospitals should establish institutional stewardship programs led by standardized national protocols for antimicrobial stewardship, restriction, and monitoring. Together, they strengthen the control ecosystem that brings into coherence legal provisions, institutional obligations, and national policy goals.

Public knowledge and professional training are also essential elements of the control framework. National campaigns focus on healthcare providers, pharmacists, and the public, stressing responsible antibiotic use and the dangers of resistance. These campaigns are coordinated through NAP and MOH so that messaging is consistent and surveillance data correspond with those reported at the national level. Education is supported by participants in the capacity-building initiatives, including infectious disease physicians, clinical pharmacists, and laboratory professionals, who are necessary to support established experts [44].

The system of control has also evolved, adapting to new challenges and international guidance. Saudi Arabia has also learnt from mass gatherings like Hajj and Umrah, where the crowd pull of the masses increases the possibility of AMR spread. Policy has become more proactive, with increased surveillance during such mass gatherings, collaboration with international health organizations, and messaging to pilgrims about the appropriate use of antibiotics. The measures depict the evolving nature of Saudi AMR policy, spanning proactive policies coupled with an evidence-based follow-up. The Saudi Arabian context and control framework for AMR is one of an integrated policy environment that encompasses multiple sectors with the amalgamation of national policy development, monitoring through regulation, stewardship at the institutional level, and awareness raising in public health terms. Efforts in the early 2010s focused on awareness and hospital-level interventions, whereas the NAP of 2017 introduced a One Health-based response that was institutionalized through subsequent updates in 2022–25 [7], 45]. The MOH, inter-ministerial committee, and National AMR Committee are the linchpins of control responsible for policy implementation, surveillance, and cross-sectoral coordination. Regulations, capacity building, and public participation contribute to the overall efficiency of the control system. Together, these endeavors create a strong foundation for the Saudi Arabian response to AMR and support pan-Saudi alignment with global plans, including the WHO Global Action Plan.

### Drivers of antimicrobial resistance (AMR) in Saudi Arabia

The development and widespread occurrence of antimicrobial resistance (AMR) in Saudi Arabia result from a complex interplay among clinical, behavioral, environmental, and systemic influences [46], 47]. Knowledge of these drivers is essential to develop effective interventions targeting the underlying factors of antibiotic resistance and not only its consequences. In the Saudi context, AMR drivers are affected by high use of antibiotics, healthcare delivery patterns, sociocultural habits and practices, as well as legislative voids, and finally ‘Saudi Arabia’s distinct identity receiving a considerable flow of visitors from around the Middle East and many global destinations [48].

The leading cause of AMR in Saudi Arabia is the high rate of antibiotic consumption; it occurs both at hospitals and at the community level. Numerous studies have shown that overall inappropriate prescribing is common, with unnecessary prescribing of broad-spectrum antibiotics for conditions not requiring antimicrobial therapy, such as viral respiratory infections, constituting a large part of the problem [6], 49], 50]. Overprescription is usually exacerbated by the empirical use of antibiotics without microbiological evidence, leading to selective pressure for resistant isolates [32]. In the community, antibiotics are occasionally bought without a prescription in contravention of laws banning over-the-counter sales. Such misuse contributes to a reservoir of resistant organisms in the general population and promotes transmission of resistance genes.

Health-service-related factors also significantly contribute to the spread of AMR. Tertiary-care hospitals in Saudi Arabia have high bed occupancy, with patients who have complicated disorders requiring extended hospital stays and antimicrobial coverage [51]. Overcrowding and rapid patient turnover are risk factors for healthcare-associated infections, which in turn often require broad-spectrum antibiotic therapy and encourage the development and spread of multidrug-resistant (MDR) pathogens. Furthermore, the inconsistent use of infection prevention and control (IPC) measures, such as adherence to hand hygiene, environmental cleaning and decontamination practices, and isolation precautions across individual hospitals, results in the nosocomial dissemination of multidrug-resistant organisms [7].

Mass gatherings are an essential and unique driver of AMR in Saudi Arabia. The annual Hajj attracts more than two million pilgrims from across the globe and creates favorable conditions for the rapid spread of communicable diseases. Both respiratory and gastrointestinal pathogens are highly prevalent in these conditions, and antibiotics are commonly used for prophylaxis or treatment during Hajj, which may accelerate the selection of resistant strains. Multidrug-resistant bacterial organisms, such as ESBL-producing *E. coli* and MRSA, have been detected in post-pilgrim returnees, representing the threat of global drug resistance spread [4], 52]. Likewise, the year-round Umrah pilgrimage heightens this threat, especially as the Kingdom continues to develop religious tourism.

There is also an international workforce, such as healthcare workers, that adds yet another layer of complexity. A reliance on expatriate healthcare workers in Saudi Arabia creates a dependence on foreign labor. These non-nationals may originate from countries with different resistance patterns and varying antimicrobial policies and practices [53]. This mobility facilitates the importation of resistant organisms into Saudi hospitals and undermines infection control and stewardship practices. Meanwhile, repatriating Saudis and expatriate residents going in the other direction will bring with them resistant infections as both sources and recipients of resistance.

AMR is, in fact, a threat to global health and development, not only as a consequence of AMR within the agricultural sector and veterinary practice. Antibiotics are used in animal husbandry for both medical and growth-promoting purposes. Regulations are in place, but enforcement is inconsistent, and antimicrobial residues can be present in food, thereby imposing selective pressure on bacterial communities of both terrestrial (human or animal) and marine origins. Environmental pollution, such as sewage from healthcare facilities and farms, acts as a reservoir of resistant genes, facilitating their horizontal transfer between bacterial populations. The One Health strategy highlights that animal and human health are interconnected, so interventions in the agricultural and veterinary sectors are needed to control AMR in Saudi Arabia [54].

AMR is also shaped by cultural and behavioral issues, based on the patient’s desire for antibiotics, particularly where respiratory illness is concerned, leaving some practitioners to feel forced to prescribe them when it is not clinically appropriate. Non-prescription use of antibiotics is prevalent in specific settings, due to lack of knowledge and hazards of inappropriate use [55]. Such behaviors exert a selective pressure for resistant variants in the community. Public campaigns have taken on these problems, but sustained shifts in behavior are proving elusive.

Surveillance and laboratory capacity Surveillance and laboratory testing, though improving, are still a driver to the extent that lack of diagnostic infrastructure has historically resulted in empiric prescribing and delays in identifying patterns of resistance [56], 57]. Hospitals with limited microbiology facilities may unnecessarily prescribe broad-spectrum antibiotics to cover the worst-case scenario, thereby promoting resistance. Therefore, the expansion of laboratory systems and the development of strong surveillance networks within health systems play a crucial role in controlling this driver. Such standardized recording of resistance patterns and antimicrobial use data supports evidence-based policy-making and stewardship interventions. The policy and regulatory deficiencies being reduced by the NAP have also historically contributed to the misuse of antibiotics in both human and animal health settings. Inadequate enforcement of the regulation prohibiting non-prescription and over-the-counter use of antibiotics, variable application of checks on antimicrobial use in veterinary settings, and insufficient incentives for hospitals to implement stewardship programs are all contributing factors to resistance rates. To address these structural gaps, the revised 2022–25 NAP seeks to respond through coordinated surveillance and stringent regulation, while embedding stewardship across all sectors, drawing on lessons from earlier policy shortfalls [58], 59]. AMR drivers in Saudi Arabia are multi-faceted and interconnected, involving clinical, behavioural, agricultural, environmental, and systemic aspects. Multiple factors, including high antibiotic use, inappropriately congested healthcare facilities, mass events, cross-border movement of human resources for health, and animal antibiotic consumption, as well as behavioral issues, contribute to the selection and spread of resistance [60], 61]. The response to such drivers needs to be a unified One Health-based approach that includes policy implementation, surveillance, stewardship programmes, public awareness, and cross-sector collaboration. These determinants are crucial for informing targeted interventions having the potential to reduce AMR sustainably and therefore safeguard public health in Saudi Arabia and beyond.

### Stewardship progress in Saudi Arabia

Antimicrobial resistance (AMR) has become a significant public health concern in Saudi Arabia, leading the Kingdom to focus on the development of Antimicrobial Stewardship Programs (ASPs) over the past 10 years. Given that the misuse and overuse of antimicrobials are increasing the incidence of multidrug-resistant organisms, Saudi health regulators have designed policies to contain and use these agents prudently across all sites of care. The country’s response mechanism is based on policy-driven programmes, institutional interventions, professional education, and surveillance activities, indicating a holistic approach consistent with international best practice.

Antimicrobial stewardship was first established in Saudi Arabia, with awareness, education, and pilot studies in large specialist tertiary care institutions, and a national antimicrobial stewardship framework in conjunction with the Gulf Arab states [7], 62]. In 2014, the Ministry of Health (MOH) established a national antimicrobial stewardship framework to promote an organized, system-wide culture of appropriate antibiotic use as part of a large regional plan involving the Arab Gulf states [7], 63]. This plan outlined an overall approach for healthcare organizations to implement stewardship activities in clinical care, focusing on responsible prescribing, tracking antibiotic use, and limiting inappropriate changes of empiric therapy. The implementation strategy has become complex, comprising interventions targeting prescriber behavior and institutional systems. Education is a cornerstone, and practicing physicians, pharmacists, and nurses need to receive evidence-based recommendations on the appropriate antimicrobial agent, its dose, and the duration of use. They also emphasise that misuse can lead to the development of resistance and promote a mindset of responsibility and choice. In addition, hospitals have established audit-and-feedback systems in which antimicrobial agents are reviewed regularly and clinicians receive feedback on their performance. Such interventions have proved effective in decreasing inappropriate antibiotic use and adherence to institutional guidelines [64].

**Figure 3:**
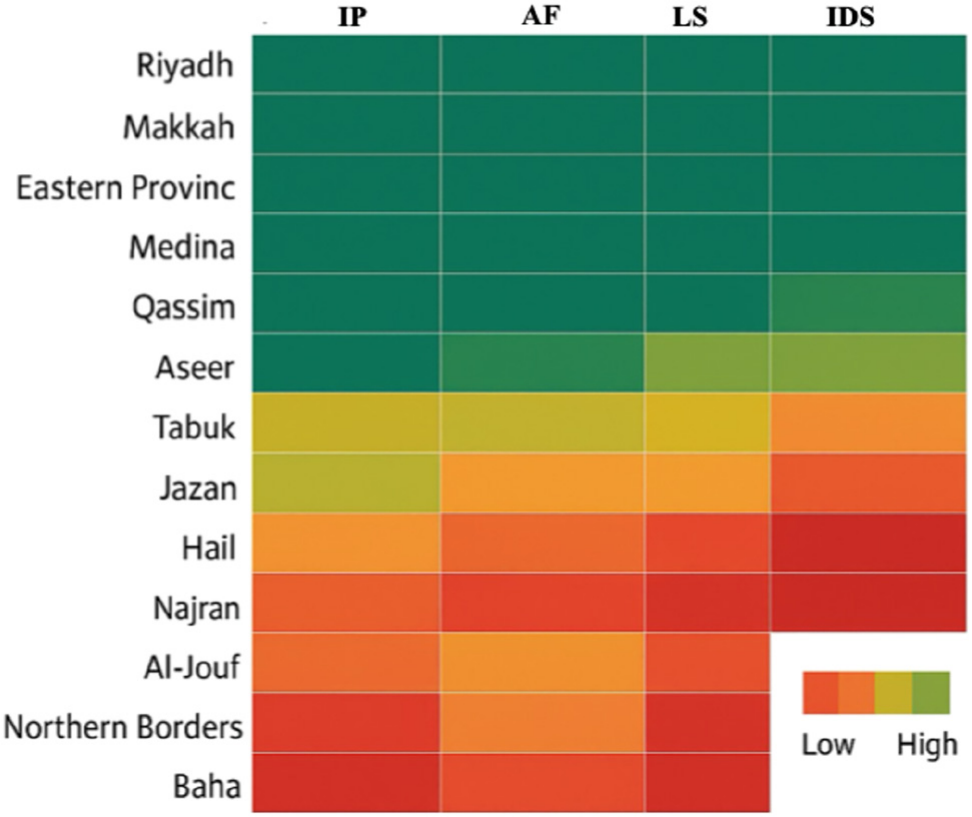
A heatmap representing the implementation of antimicrobial stewardship programs (ASPs) across healthcare regions in the Kingdom of Saudi Arabia in 2025. The heat map shows the level of ASP adoption across the 13 health regions in Saudi Arabia, based on a composite index (IP, institutional presence; AF, audit-feedback; LS, laboratory support; and IDS, infectious disease physician availability). Color gradation: Dark green=high (≥70 %), Yellow=Moderate (40–69 %), Red=low (<40 %).

Other stewardship interventions include intravenous‐to‐oral switch programs, restrictive policies on high-risk or broad-spectrum antimicrobials, and hospital-specific treatment guidelines. Intravenous-to-oral antibiotic switch also minimizes the risk of nosocomial complications and economizes resources by shortening the length of stay and reducing drug expenditures [65]. Restriction policies, which mandate pre-authorization or post-prescription review of specific antimicrobial agents, have been particularly successful at reducing inappropriate utilization of carbapenems, vancomycin, and other key agents [66]. The use of these protocols to standardize prescribing reduces selective pressures that promote resistance evolution within the hospital. These stewardship efforts have produced measurable and hopeful results. Studies spanning the entire nation and multicenter studies show a decrease in overall antimicrobial use after the implementation of antimicrobial stewardship programmes (ASPs), especially at teaching hospitals with well-developed programs [67]. Surveillance data have shown decreases in some key multidrug-resistant pathogens, including extended-spectrum beta-lactamase (ESBL)-producing *E. coli* and methicillin-resistant *S. aureus* (MRSA), indicating positive directional trends associated with stewardship interventions [68], 69]. The distribution and intensity of stewardship implementation across Saudi health institutions are illustrated in Figure 3. The highest levels of implementation are in Riyadh (90 %), Makkah (85 %), and the Eastern Province (82 %), while the lowest coverage is observed in Northern Borders, Baha, and Najran (≤45 %). The national average ASP coverage is approximately 68 percent.

In addition, clinical results have been enhanced by earlier hospital discharge, fewer healthcare-associated infections, and more effective use of resources, with significant improvement in antimicrobial guideline compliance after the induction of stewardship, leading to better patient care and earlier use of hospital resources [70]. Yet, there are still challenges to the complete implementation of stewardship programs in all healthcare settings. Resource limitations, including a lack of access to infectious disease specialists, clinical pharmacists, and trained microbiologists, are barriers to program expansion, particularly at smaller or rural hospitals. Knowledge and educational gaps persist regarding HCWs, warranting ongoing professional development to support stewardship practice. Moreover, differences in electronic health record infrastructure and functionality for data collection make the real-time monitoring of antimicrobial prescribing and assessment challenging, hindering the provision of rapid feedback and the optimization of programs [71], 72]. These challenges illustrate the importance of continued investment in human capital, technological infrastructure, and country-wide training programs to sustain stewardship efforts. These challenges have been addressed at the national level by incorporating stewardship into the National Action Plan (NAP) on AMR [38]. In the 2017 NAP, commensurate with the World Health Organization’s Global Action Plan, five key areas were identified for achieving optimal antimicrobial use: creating greater awareness, including multi-sectoral advocacy; establishing stronger multidrug resistance surveillance; promoting cutting-edge research; and reinforcing institutional capacity [73]. Revisions made in 2022-5 broadened the scope of stewardship beyond tertiary hospitals to include community health care sites, veterinary practices, and agricultural establishments, embracing a One Health perspective. The revised NAP emphasises integrated surveillance, policy implementation, and cross-sectoral collaboration as essential in driving sustainable reductions in antimicrobial misuse [54].

The incorporation of technology is another potential avenue for stewardship advancement. The use of electronic health records (EHRs), clinical decision support tools, and automated surveillance systems has the potential to enable real-time monitoring of antibiotic use, identify trends in inappropriate prescribing practices, and offer clinicians evidence-based recommendations at the point of care [74]. Such digital aids can not only improve program efficiency but also make data collection systematic for policy decisions and targeted interventions. Stewardship still includes engaging with the public and educating them. These campaigns hope to instill a warning into the population about the potential risks of self-medication and also taking antibiotics exactly as prescribed. These programs can augment hospital-based stewardship efforts by addressing community-induced misuse, a major driver of resistance, and bolstering research and surveillance to serve stewardship goals. In Saudi Arabia, molecular epidemiology, resistance mechanisms, and the effect of stewardship interventions on clinical outcomes are becoming areas of interest [75], 76]. Developing national centralized data repositories and connecting regional networks of laboratories will provide more accurate surveillance of the direction and rate of AMR trends and support evidence-based decision-making for stewardship policy and practice. Saudi Arabia has demonstrated substantial achievements in promoting antimicrobial stewardship during the last 10 years. Incorporation of ASPs into hospital systems, facilitated by national policies, education, and surveillance, has led to enhanced antimicrobial practices, decreased resistance rates, and improved patient outcomes [77]. Although challenges in resource and data infrastructure and provider education remain, planned initiatives under the NAP and One Health concepts offer a strong foundation for continued progress. Continued dedication to improving antimicrobial stewardship through innovation and cooperation between sectors will be needed to guarantee the future success of AMS programs in Saudi Arabia and to help secure the effectiveness of antimicrobial agents for generations to come.

### Molecular mechanisms to control antimicrobial resistance across different sectors

Beyond policy and stewardship interventions, molecular approaches are increasingly important for controlling antimicrobial resistance across the human, animal, and environmental sectors. The control of AMR across human, animal, and ecological sectors  now depends on molecular methods, which facilitate early diagnosis of resistance, targeted intervention, and the breaking of transmission chains [78]. In human medicine, molecular diagnostics, including PCR-based assays, multiplex panels, and whole-genome sequencing, have enabled rapid detection of resistance genes and mutations, thereby promoting precision antimicrobial therapy whilst minimizing unnecessary exposure to broad-spectrum agents [79]. Genomic epidemiology additionally benefits outbreak investigation by providing evidence of clonal spread and transmission pathways within institutions, thereby supporting rapid infection prevention and control interventions [80]. In veterinary and farming medicine, molecular monitoring of resistance determinants in food animals, animal-derived food products, and farm environments can identify sources of resistance and support the prudent use of antimicrobials [81]. Monitoring of transferable resistance genes enables focused regulatory interventions, encourages stewardship in animal health, and facilitates assessment of alternatives, such as vaccination and biosecurity [82]. Environmental molecular surveillance, including wastewater and food systems, is essential for detecting resistance genes circulating in the nonclinical setting. Metagenomics can provide information on the resistome and mobilome, identifying hotspots for horizontal gene transfer and environmental amplification of resistance. Combining molecular data from human, animal, and ecological sources within national surveillance systems can support early warning, facilitate risk assessment, and prompt coordinated sectoral responses. Together, molecular tools contribute to evidence-based stewardship, regulatory decisions, and operations for One Health approaches targeting the emergence and dispersal of antimicrobial resistance.

### Hospital-based infection prevention and control measures

Given that hospitals serve as primary reservoirs of antimicrobial-resistant pathogens, robust infection prevention and control measures are essential to limit transmission. The majority of resistant pathogens originate in and spread through hospitals, especially high-risk areas such as intensive care units (ICUs), surgical wards, or extended-care facilities [83]. Hence, good infection prevention and control (IPC) practices play a significant role in preventing the development and spread of resistant organisms within health care settings. Fundamental IPC measures include rigorous hand hygiene, which remains the first and most important measure to prevent healthcare-associated infection. Regular environmental cleaning and disinfection of areas surrounding patient care, medical devices, and high-contact surfaces is vital to reduce environmental reservoirs of resistant bacteria.

Early detection and management of colonized or infected patients through active surveillance, admission screening for high-risk individuals, and effective isolation or cohorting measures are essential interventions, especially for multidrug-resistant organisms (MDROs) such as carbapenemase-producing Enterobacteriaceae (CRE), methicillin-resistant *S. aureus* (MRSA), and multidrug-resistant *A. baumannii* [84]. Use of device-related infection prevention bundles for central lines, urinary catheters, and mechanical ventilation decreases the risk of pathogen invasion and, with it, the risk of subsequent antibiotic exposure.

A combination of IPC measures with antimicrobial stewardship programs will also improve effectiveness by reducing unnecessary antibiotic use, which fuels selective pressure in hospitals. Ongoing education and training of healthcare workers, adherence measurement, and audit-and-feedback strategies are necessary to maintain IPC measures. This set of coordinated hospital-based interventions in IPC will help reduce healthcare-associated infections, break the chain of transmission, and is crucial for controlling AMR in the clinical care setting.

### Roadmap for enhancing antimicrobial resistance control in Saudi Arabia

The rising threat of antimicrobial resistance (AMR) in Saudi Arabia demands a comprehensive, multisectoral, and evidence-based roadmap to guide national action to prevent, contain, and mitigate it. Drawing on the NAP for AMR, 2022–2025 [38] and aligned with WHO’s Global Action Plan on AMR [10], the roadmap centers on four interconnected pillars: strengthening surveillance, leveraging antimicrobial stewardship, improving regulatory and policy environments, and stimulating research and innovation. These pillars are designed to collectively tackle the causes of AMR and introduce sustainable, scalable interventions across human, animal, and environmental health with a One Health approach.

#### Strengthening surveillance systems

Successful AMR containment starts with an all-inclusive, timely surveillance. The roadmap underscores the need to expand laboratory facilities, standardize diagnostic procedures, and integrate hospital, community, veterinary and environmental surveillance systems. Surveillance should consider not only resistance trends but also patterns in antimicrobial use on a sector basis. The establishment of national databases on antimicrobial use and resistance would allow prompt detection of emerging hotspots of resistance, facilitating targeted interventions. As noted for mass gatherings such as Hajj and Umarah, improved sentinel surveillance is essential for detecting and controlling multidrug-resistant organisms, thereby reducing national and international spread [4]. Electronic health records and integrated laboratory reporting systems are digital platforms that can facilitate real-time data collection, analysis, and dissemination to help guide policy-maker and clinician decisions.

#### Optimizing antimicrobial stewardship

One keystone of the roadmap is to roll out and standardize ASPs in all health institutions throughout the country. Such stewardship should not be limited to tertiary hospitals and should extend to primary care centers, community clinics, and private practices. The roadmap advises that institutional ASPs should be formalized within the structure of precise control, specific goals, and accountability. Significant interventions include guideline-based prescribing, audit and feedback, restriction policies on particular antibiotics, and education for clinicians and pharmacists. Application of digital decision-support systems may further enhance prescription accuracy and protocol adherence. In Veterinary Medicine and Agriculture, stewardship schemes should target optimizing antibiotic use, reducing non-therapeutic use, and encouraging viable alternatives, such as vaccination and biosecurity strategies [85], 86]. The standardization of stewardship across the human, animal, and environmental health sectors can reduce the selective pressures that contribute to resistance in Saudi Arabia, while enabling the sustainable use of antimicrobial agents [7].

#### Enhancing regulatory and policy frameworks

Regulation, however, is among the key enablers of AMR control [87]. The roadmap calls for rigorous enforcement of antibiotic prescription-only on both the human and animal sides, backed by regular inspections and the Saudi Food and Drug Authority. Policies must cover pharmaceutical production and distribution channels, quality control of imported drugs, and regulation of antimicrobial applications in livestock. The roadmap emphasises the need to mainstream AMR control in the overall health systems plan, calling for inter-ministerial coordination among the MOH, the Ministry of Environment, Water and Agriculture, and other relevant authorities. Furthermore, public health campaigns should further sensitise communities on how to use these antimicrobials and stress the risks of self-medication and of not completing treatment regimens. Domestic policy frameworks must be flexible enough to change, responding to lessons from surveillance data, international best practices, and the emergence of resistance patterns.

#### Promotion of research, innovation, and capacity development

Effective measures to combat AMR are underpinned by research and innovation [88]. The roadmap target identifies investment in epidemiological studies, molecular characterisation of resistance mechanisms, and evaluation of stewardship interventions. The research needs to prioritize the country’s needs, including studying AMR in the community as well as in hospitals; evaluating resistance dissemination due to mass gatherings; and elucidating the role of the environment and agriculture sectors in resistance propagation. Strengthening of capacity in microbiology, infectious diseases, pharmacy, and epidemiology is necessary to support surveillance, clinical decision-making, and research. Academics, regional centers of excellence, and international bodies may establish partnership programs for knowledge transfer, technical support, and collaborative research. Furthermore, support for the development of regional rapid diagnostic tools, vaccines, and new antibacterial agents will enhance the Kingdom’s ability to respond to emergent resistant pathogens.

#### Integration of One Health principles

The roadmap focuses on translating One Health approaches and functions, including the interactions among human, animal, and environmental health, to contribute to AMR [89]. Synchronized surveillance of antibiotic use in livestock, wastewater, and food production systems is also crucial for avoiding the environmental spread of resistant bacteria. Cross-sectoral communication tools need to be put in place to share data for surveillance, harmonize interventions, and respond quickly to evolving resistance threats. Utilizing integrative One Health approaches can also help Saudi Arabia anticipate AMR reservoirs before they enter clinical settings.

#### Implementation and evaluation

Structured control and performance appraisals are necessary to manage the roadmap effectively. Oversight at the national level by both the MOH and the inter-ministerial AMR committee provides for coordinating compliance with NAP and international standards. Performance metrics should be defined to track changes, including in rates of inappropriate antimicrobial use, prevalence of key resistant pathogens, adherence to stewardship protocols, and reach of public education efforts. Ongoing monitoring and reporting will enable continuous quality improvement, support policy modification, and ensure accountability. For broad ownership and continuity of implementation, multi-stakeholder involvement, including the health, agriculture, environment, and academia sectors, is indispensable.

#### Prioritizing high-risk settings

Priority interventions in high-risk settings-such as ICUs, tertiary hospitals, long-term care facilities, and mass gathering venues- are also outlined in the roadmap. Strengthening infection prevention and control (IPC) measures, rapid diagnostics, and stewardship programmes in these settings can diminish the burden of resistant infections and prevent further transmission. Mass gathering policies may include pretravel screening, vaccination programs, health education and hygiene promotion, and a specific focus on surveillance for multidrug-resistant organisms among pilgrims.

#### Sustainability and future directions

The roadmap is based on the principle of sustainability. Long-term investments are needed in human resources, laboratory capacity, digital health systems, and public engagement to avoid backsliding. AMR controls can be mainstreamed into national health priorities and annual fiscal programming to facilitate systematic resource allocation. Moving forward, Saudi Arabia plans to leverage artificial intelligence, big data analytics, and genomic surveillance to improve predictive ability and precision in AMR interventions. The foundation supports a proactive, anticipatory model that can detect and contain new resistance threats at an early stage, and a commitment to facilitating broad agreement on global goals for the containment of AMR.

Saudi Arabia’s systematic EOL for AMR control provides a detailed, all-inclusive, and evidence-based outline in accordance with the NAP (2022–2025) and the WHO Global Action Plan [10], 38]. By improving surveillance, promoting stewardship, building regulatory environments, stimulating research and innovation, and translating One Health into action, the roadmap offers a tangible approach to fight back against AMR. Comprehensive adoption, ongoing monitoring, and sustained commitment at the intersectoral level will be necessary to maintain the efficacy of antimicrobials and protect public health in the Kingdom and region. Fundamental actions, implementation pillars, and monitoring indicators for AMR containment are outlined in Table 2.

**Table 2:** Saudi Arabia’s roadmap for antimicrobial resistance containment (2022–2025): pillars, key actions, KPIs, and references. The roadmap aligns with the National Action Plan (NAP 2022–2025) of Saudi Arabia and the WHO Global Action Plan. Indicators and targets are based on established national priorities and the available global AMR surveillance frameworks.

Pillar	Strategic actions	Key performance indicators (KPIs)	Target by 2025	Reference
Surveillance strengthening	Expand national laboratory network; standardize AMR data reporting (human, animal, environmental).	≥90 % hospitals integrated into national AMR system; quarterly data publication.	≥90 %	[90]
Stewardship implementation	Universal ASP rollout in all MOH hospitals; training of clinicians and pharmacists.	≥80 % facilities with active ASP teams; ≥70 % compliance with prescribing guidelines.	≥80 %	[91]
Regulation and governance	Enforce prescription-only antibiotic sales; strengthen veterinary regulation.	>95 % pharmacies compliant with over-the-counter restriction.	>95 %	[10]
Research and innovation	Fund molecular epidemiology, AI-enabled diagnostics, and vaccine R and D.	≥15 research grants funded; ≥2 national innovation pilots launched.	≥15 grants	[92]
One Health and public engagement	Cross-sector AMR committee; nationwide public awareness campaigns.	Annual campaign reach >10 million; inclusion of One Health indicators in reports.	>10 million people reached	[93]

AMR, antimicrobial resistance; ASP, antimicrobial stewardship programme; KPI, key performance indicator; MOH, Ministry of Health; R&D, research and development.

#### One Health-based prevention strategies

Combating AMR demands a combined One Health approach that addresses human, animal, and environmental drivers simultaneously. Enhanced antimicrobial stewardship in both human and veterinary medicine is crucial. It comprises evidence-based prescribing, restricting broad-spectrum agents, audit-and-feedback systems, rapid diagnostics for targeted therapy, prescription-only access measures, vaccination, and biosecurity improvements in animal health. Similarly, greening and regulating the use of antibiotics in agriculture/food production are significant, primarily by restricting non-therapeutic use, enforcing residue levels, monitoring antibiotic consumption, and tracing back through the food chain. Environmental surveillance, wastewater, and food-chain monitoring provide early signals of resistance emergence and dissemination beyond the clinical environment, identifying reservoirs for horizontal gene transfer. Combining human, veterinary, agricultural, and environmental surveillance into harmonized nationwide systems enables real-time exchange of information for evidence-based, joined-up responses. Together, these integrated strategies reduce selective pressure on resistance, break the transmission cycle, and facilitate sustainable national and international actions to address AMR.

### Expert opinion and future perspectives

The fundamental Messages about Antimicrobial resistance (AMR) in Saudi Arabia present a multifaceted public health challenge that demands sustained, multifactorial, and long-term interventions. Despite significant advancements made in the previous decade, including the issuance of a national action plan (NAP 2022–25), the institutionalization of antimicrobial stewardship programs (ASPs), and the expansion of surveillance networks, resistance rates continue to endanger patient care, financial sustainability, and regional public health protection. The expert view is that a paradigm shift is needed: incorporating sound control, technological innovation, multisectoral collaboration, and public participation to respond to short- and long-term drivers of resistance. Finally, from a clinical point of view, precision stewardship and tailored antimicrobial treatment have to be underlined by experts. Empirical Antibiotic use based on noisy data in traditional empiricism, which is widely practiced in many healthcare facilities, leads to the emergence and selection of multidrug-resistant organisms. Novel treatment modalities in the future should incorporate rapid diagnostic tests, molecular diagnostics, and susceptibility profiling to direct therapy on enrollment. Near-patient diagnostics, together with electronic decision support, can minimise empiric broad-spectrum antibiotic courses, optimise therapy duration, and reduce the development of resistance. The Kingdom of Saudi Arabia, with its investment in digital health and smart hospital infrastructure, is optimally situated to adopt those technologies at scale.

At the policy and control levels, professionals argue for further reinforcement of the One Health approach. AMR is undoubtedly a multisectoral issue; hence, multidisciplinary approaches of human, animal, and environmental health are crucial. This includes implementing regulations on the use of veterinary antimicrobials, conducting residue surveillance in food products, and preventing pollution through improved waste management. In addition, there is expert agreement that closer ties with foreign partners, and above all with Saudi Arabia as a pilgrimage and medical tourism destination, are necessary. Joint surveillance with feeder and host countries will involve early identification of imported resistant strains and expedited containment response.

A crucial future direction is to strengthen research infrastructure and translational science for national-level interventions. While there have been improvements in surveillance and stewardship, essential gaps persist in the molecular epidemiology of resistant pathogens in Saudi Arabia. Authorities stress the need for research to focus on high burden pathogens, such as ESBL-producing Enterobacteriaceae, carbapenem-resistant *A. baumannii*, and MRSA. Genomic sequencing, bioinformatics, and phylogenetic analyses can uncover resistance mechanisms, transmission chains, and outbreak sources. These insights will inform evidence-informed policy making by helping to design targeted interventions, estimate the risks of new threats, and use resources effectively.

Education and changing behaviour remain a mainstay of expert advice. Public and professional education campaigns should move beyond simple messages to consider the sociocultural drivers that affect antimicrobial usage. Guidelines, prescription habits, and interpretation of susceptibility data should be the main points addressed in continuous training for physicians. At the public level, campaigns should concentrate on decreasing self-medication, improving adherence to prescribed treatment, and stressing the collective societal consequences of AMR. Encouraging an attitude of antimicrobial prudence is as vital in limiting drug resistance as formal rules or technological interventions.

Experts also refer to the challenge and opportunity presented by mass gatherings, in particular, the Hajj and Umrah. Such events increase exposure and thus the risk of AMR dissemination due to the large number of foreign visitors, while also providing a managed setting for pilot interventions. Pretravel vaccination, focused screening, and post-pilgrimage monitoring are examples of approaches that can be models for large-scale, evidence-based AMR control. Incorporating lessons learned from mass-gathering health management into our routine public health planning can improve the Kingdom’s preparedness for emerging infections and resistance containment.

Digital health and artificial intelligence (AI) are increasingly recognized as essential contributors to the future of AMR control. AI-powered analytics can forecast resistance trends, optimize hospital formulary management, and support outbreak monitoring. Machine learning algorithms, across electronic medical records and laboratory results, can recognize prescribing habits, highlight high-risk cases, and recommend alternative treatments that a stewardship program would also endorse. Continuing investment by Saudi Arabia in digital infrastructure means that the country is well placed to utilise these tools effectively, building up predictive analytics for clinical and public health interventions.

There’s also an emphasis from experts on sustainability and long-term resilience. AMR containment is not a “quick fix,” but an ongoing effort that requires continual input of human resources, laboratory capacity, and regulatory compliance, not to mention public awareness. The progress on AMR needs to be ‘hardwired’ into long-term health systems strengthening efforts so that it survives changes of government and remains unaffected by policy fluctuations, system stresses, or economic challenges. To sustain momentum and monitor impact in the long term, multi-sector coordination, assured funding streams, and strong monitoring systems are essential.

Lastly, Saudi Arabia has the potential to be a regional AMR leader with neighboring Gulf and broader Middle Eastern countries following its lead. A great opportunity exists to showcase scalable AMR containment models due to the Kingdom’s healthcare system capabilities, research investments, and centralised control. Publishing results, data sharing with regional networks, and participating in collaborative research would also help to build up local knowledge and contribute to regional health security. The road to controlling AMR in Saudi Arabia depends on combining integrated, forward-looking elements, including precision medicine and One Health coordination, with robust research, technological innovation, and sustained behavioral change. Expert opinion emphasizes that significant progress has been made, but ongoing vigilance, prudent investment, and effective control are needed to ensure the continued effectiveness of antimicrobials. If these guidelines are applied, Saudi Arabia can reduce the menace of AMR and enhance its ability to respond to public health challenges and to steward antimicrobials at national, regional, and international levels.

## Conclusions

The issue of antimicrobial resistance is complex and a moving challenge in Saudi Arabia, one that necessitates unified, evidence-based, and long-term efforts. While the country has made good progress in the National Action Plan, the Antimicrobial Stewardship Program, and regulatory interventions, the resistance profile remains high across multiple pathogens. Responding to this challenge will involve strengthening surveillance capacity, diagnostic capability, and behavioral interventions. One Health concepts, promoted among humans, animals, and the environment, will be necessary for long-term control. Utilisation and surveillance of antibiotics can be optimised further through digital health tools and artificial intelligence. Sustained policy commitment, cross-sector collaboration, and support for research and training are essential to achieving sustainable success. Through effective leadership in stewardship and surveillance, Saudi Arabia can preserve the efficacy of antimicrobials within its borders and implement actions proposed to contain AMR in the region and globally.
